# A cluster randomised trial testing an intervention to improve parents’ recognition of their child’s weight status: study protocol

**DOI:** 10.1186/s12889-015-1882-3

**Published:** 2015-06-12

**Authors:** Kathryn N. Parkinson, Angela R. Jones, Martin J. Tovee, Louisa J. Ells, Mark S. Pearce, Vera Araujo-Soares, Ashley J. Adamson

**Affiliations:** Institute of Health & Society, Newcastle University, William Leech Building, Newcastle upon Tyne, NE2 4HH UK; Human Nutrition Research Centre, Newcastle University, Newcastle upon Tyne, UK; Institute of Neuroscience, Newcastle University, Newcastle upon Tyne, UK; Health and Social Care Institute, Teesside University, Middlesbrough, UK

**Keywords:** Obesity, Overweight, Children, Parents, Body size perceptions, Weight status, Intervention

## Abstract

**Background:**

Parents typically do not recognise their child’s weight status accurately according to clinical criteria, and thus may not take appropriate action if their child is overweight. We developed a novel visual intervention designed to improve parental perceptions of child weight status according to clinical criteria for children aged 4–5 and 10–11 years. The Map Me intervention comprises age- and sex-specific body image scales of known body mass index and supporting information about the health risks of childhood overweight.

**Design:**

This cluster randomised trial will test the effectiveness of the Map Me intervention. Primary schools will be randomised to: paper-based Map Me; web-based Map Me; no information (control). Parents of reception (4–5 years) and year 6 (10–11 years) children attending the schools will be recruited. The study will work with the National Child Measurement Programme which measures the height and weight of these year groups and provides feedback to parents about their child’s weight status. Before receiving the feedback, parents will complete a questionnaire which includes assessment of their perception of their child’s weight status and knowledge of the health consequences of childhood overweight. The control group will provide pre-intervention data with assessment soon after recruitment; the intervention groups will provide post-intervention data after access to Map Me for one month. The study will subsequently obtain the child height and weight measurements from the National Child Measurement Programme. Families will be followed-up by the study team at 12 months. The primary outcome is any difference in accuracy in parental perception of child weight status between pre-intervention and post-intervention at one month. The secondary outcomes include differences in parent knowledge, intention to change lifestyle behaviours and/or seek advice or support, perceived control, action planning, coping planning, and child weight status at 12 month follow-up.

**Discussion:**

The Map Me tool has potential to make a positive impact on children’s health at a population level by introducing it into current intervention programmes to improve accuracy of parental perception of child’s weight status. This trial will inform the action of researchers, educators, health professionals and policy makers.

**Trial registration:**

Current Controlled Trials ISRCTN91136472. Registered 3 May 2013

## Background

The prevalence of overweight and obesity in childhood is a public health concern for the United Kingdom [1]. To effectively combat this problem, the emphasis needs to be placed on population-wide prevention in a range of settings including the home environment [1]. Parents play an important role in shaping children’s health related behaviours [2] and are relied upon to recognise unhealthy weight gain and take the necessary action [3]. However, there is a growing evidence base demonstrating that parents of overweight and obese children typically do not describe their child as such [3–7].

Data on how parents’ perceptions of their child’s weight status can be improved are limited. The National Child Measurement Programme (NCMP) currently measures height and weight of school children in reception (age 4–5 years) and year 6 (age 10–11 years) in maintained schools, that is all but special needs and fee-paying schools, in England. These data are used to calculate each child’s body mass index (BMI): an index of overall body fat based on height and weight (BMI = weight[kg]/height^2^[m]). The BMI scores are converted to age and sex specific centiles calculated using the British 1990 growth reference UK90 [8] to determine the number of children defined as underweight, healthy weight, overweight or obese as a proportion of the number measured. Children are classified as ‘underweight’ if they have a BMI below the 2^nd^ centile; as ‘healthy weight’ between the 2^nd^ and 90^th^ centiles; as ‘overweight’ between 91^st^ and 97^th^ centiles; and as ‘very overweight’ at or above the 98^th^ centile. These results are typically communicated to parents within six weeks of measurement by letter which informs them of their child’s BMI and the UK90 category into which this falls, usually with a Change4Life leaflet (a public health programme to tackle obesity in England, and run by the Department of Health) and information on local services. Evaluations of the letters indicate that they are often not well received by parents of overweight children who doubt the veracity of the assessments, and therefore may fail to promote appropriate action [9, 10]. Similarly, the growth chart is regularly used to monitor children’s growth and to educate families about the growth process. However, the usefulness of these charts depends on whether parents are able to understand them and existing evidence shows that the general population does not [11]. Other approaches may therefore be more effective than teaching parents mathematical concepts [12].

Parents do use subjective approaches to evaluate their child’s weight status [5]. They report making visual comparisons within peer groups and tend to rely on extreme cases as a reference point [6]. As the population becomes increasingly overweight, an upward shift in the threshold for perception of child overweight may have occurred [13]. When parents are asked to classify their child’s weight status, BMI is a significant predictor, but visual representations of body fatness such as skinfolds and waist circumference contribute most to their responses [14]. Given parents’ reliance on visual characteristics [6, 14], it may prove beneficial to use visual images of child weight status to improve parents’ identification of overweight in their children. Some research has explored parental perceptions of child weight status using visual images [15–18], but the methods used are limited because they are not usually based on BMI. Even those that are based on BMI are limited. For example, the Children's Body Image Scale consists of photographic figures of pre-pubescent children, seven each of boys and girls [19]. The children range from very thin to obese and each figure corresponds to a known BMI range covering the 3rd to 97th BMI percentiles for 10-year-old children based on United States’s standards, the National Center for Health Statistics [20]. None exist which include the full range of BMI measured in English children and correspond to commonly used UK90 [21] criteria for overweight in England.

Once parents recognise that their child is overweight, parents can play a positive role in the further development of their child’s weight in two ways: they can either seek external support for weight control (such as those provided by local services), and/or they can take action at home by changing aspects of their child’s diet, time spent in sedentary or physical activities. Previous research has explored factors associated with parents’ readiness to modify the lifestyle of their overweight child; parents’ perception that their child’s weight was a health problem was associated with an increased likelihood of them implementing lifestyle changes [2]. Providing parents with information about the health risks and consequences of childhood overweight to encourage positive changes in their child’s life should, therefore, be beneficial. However, recognition and knowledge alone do not guarantee action. There is considerable evidence showing that social cognitions, such as those postulated by the Theory of Planned Behaviour [22] and Social Cognitive Theory [23], are important predictors of health-related behaviours. Both theories emphasise the key role of intentions to act or behavioural goals and of self-efficacy (the perceived capability of performing the target behaviour), for example, the ability to make a difference to the child’s weight. In addition to knowledge, intentions and goals are also a reflection of the attitudes people hold towards the behaviour (evaluation of favourableness based on specific expected outcomes) and the subjective norm. While these social cognitions are predictive of health-related behaviour [24] there is increasing recognition that people do not always act in accordance with their intentions and beliefs. This phenomenon has been called the intention-behaviour gap [25]. To bridge this gap, more recent theories such as the Health Action Process Approach [26] incorporate additional, self-regulatory constructs, such as action planning and coping planning which have improved the prediction of behaviour [27]. By specifying when, where and how to act (action planning) and how to respond to certain obstacles and barriers to action (coping planning), individuals become more likely to enact their intentions [28–30].

This trial will measure parental perception of their child’s weight status, knowledge of the consequences of a child being overweight and intention to change lifestyle behaviours and/or seek advice. Intention will be assessed by items defined by the Target, Action, Context and Time principle [31]. The study will also measure predictors of behaviour change in parents including: perceived control over child’s behaviour and self-efficacy defined as the parent's confidence in performing a behaviour [32, 33]; action planning [32, 34]; coping planning, a barrier-focused strategy to avoid unwanted responses to situational demands or social pressure [35] and measures of parent-reported behaviours aimed at seeking support and facilitating weight control in their children [36].

The aim of the trial is to test whether, compared to controls, providing the Map Me intervention [37] to parents:

- increases the proportion of parents accurately assessing their child’s weight status (convergent with the UK90 criteria [21])
- increases parental knowledge of the consequences of their child being overweight
- improves parental intention to take action, perceived control over child’s behaviour and self-efficacy, action planning and coping planning
- is associated with weight status outcomes in children at 12 month follow up

Additionally, differences in the effects of using the web-based and paper-based formats will be investigated.

## Methods

### Study design

**Fig. 1:**
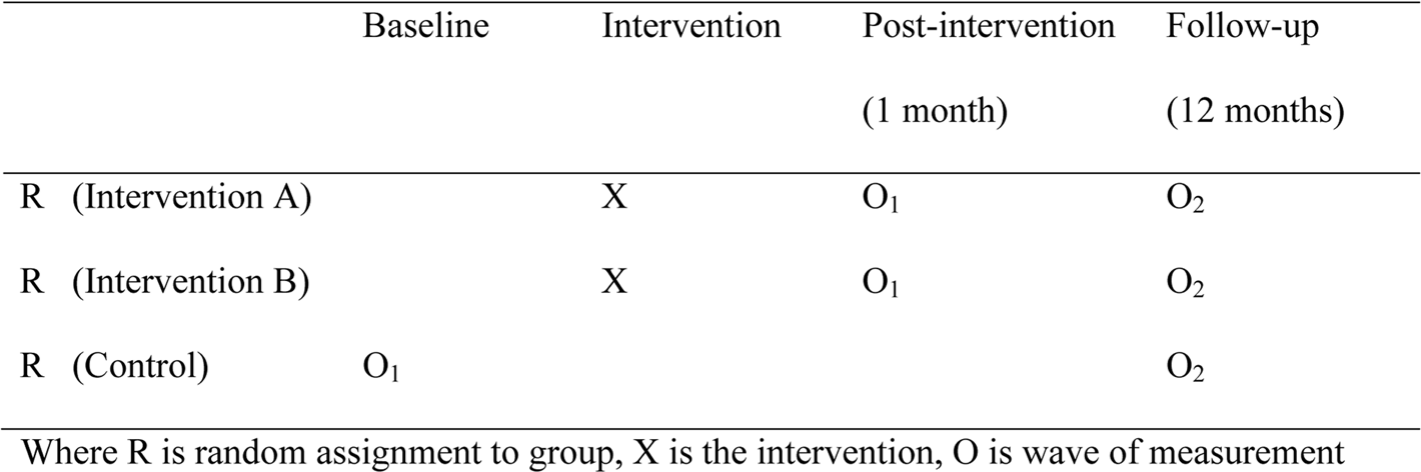
Study design

A three arm cluster randomised trial with primary schools as the unit of randomisation will be conducted with a minimum of 36 schools. It will test two versions of the intervention against the control group (Fig. 1). The intervention period is four weeks, and follow-up is 12 months.

### Intervention

This research team has developed visual age and sex-specific body images scales; there are four scales (girls 4–5 years; boys 4–5 years; girls 10–11 years; boys 10–11 years). Each scale consists of images of body shapes of known BMI ranging from underweight to very overweight in the following categories: underweight; lower healthy weight; mid healthy weight; upper healthy weight; overweight; very overweight (clinically obese); very overweight (extremely obese). Supporting information was also developed about the health risks of childhood overweight, healthy lifestyle behaviours and sources of support and advice (for example: general practitioner or school nurse; National Health Services (NHS); weight management programmes for children). The body image scales and supporting information are combined to form an intervention called ‘Map Me’. Map Me has been developed in two formats:

#### Intervention A

A parent information pack containing a paper-based version of Map Me appropriate to their child (i.e. age and sex specific).

#### Intervention B

A parent information pack with the website address and login details for the web-based version of Map Me.

Depending on randomisation, parents in the intervention groups will have *either* the opportunity to read the paper-based Map Me which includes body image scales with the weight status of each image indicated (for example, underweight, healthy weight, overweight) *or* access to an interactive web based tool utilising the body image scales with facility for the parent to choose the body image they think best represents their child, this image will then shrink or expand to the correct representation when the child’s height and weight are imputed, and a facility which will allow the child’s correct body image to be projected forward to predict their future adult body image assuming their current weight status tracks into adulthood. In this method of delivery, appropriate feedback on their child’s BMI is given. Both methods provide information about the consequences of childhood overweight. The development of Map Me will be published in full in a separate paper.

### Procedure

Within schools, all the parents of 4–5 and 10–11 year old children will be sent an invitation to join the study. Recruited parents will be those who return a signed consent form. Every school that has at least one consented child will be randomised to Intervention A, Intervention B or control using a 1:1:1 ratio, as described in the ‘randomisation of schools’ section.

Parents in the control group will provide the baseline data for the trial. They will not receive either intervention during the study period. They will be posted the study questionnaire soon after the study team receive their consent forms; the questionnaire consists of the outcome and explanatory measures. Parents in the intervention groups will receive the intervention by post. Those randomly assigned to the web-based version will additionally receive a follow-up email with website link, if an email address was obtained. The intervention period lasts four weeks and after this period has elapsed all parents in the intervention groups will be posted the study questionnaire. Parents will be asked to complete and return the questionnaire to the study office in the provided postage-paid envelope. In the majority of cases, the study team will obtain the child height and weight measurements from the NCMP. All parents and children will be followed up at 12 months after the first day of the intervention; parents will be sent the study questionnaire to complete and return to the study office, and the children will be measured for height and weight by the study team, usually at school.

A favourable ethical opinion was obtained from National Research Ethics Service, Committee North East - Newcastle and North Tyneside 2: reference 12/NE/0409.

### School sample

Schools will be drawn from Local Education Authorities (LEA) regions in England. Regions will be selected by the study team based on knowledge of the schedule of the NCMP. Special needs and fee-paying schools will be ineligible for inclusion in the trial, to align with the NCMP mandated in LEA-maintained schools only. The body image scales included in the Map Me tool were developed using a primarily Caucasian sample of children; therefore schools with over 35 % Black and Minority Ethnic population will not be included. All other schools will be invited and those agreeable will be enrolled to the study. In some cases local authorities may offer to send the study invitation letters directly to parents, eliminating the need to enrol the school.

### Randomisation of schools

The 2010 Index of Multiple Deprivation (IMD) [38] is one of the most commonly used indicators of deprivation and the one used in the NCMP. It combines a number of indicators, chosen to cover a range of economic, social and housing issues, into a single deprivation score for each small area in England. Schools will be stratified into low, medium and high IMD tertiles (based on school postcode) by the study team. These data will be provided to the Newcastle Clinical Trials Unit and a statistician, independent to the study team, will randomly allocate the stratified schools in a block design.

### Measures

Parental assessment of their child’s weight status

- questionnaire item: ‘How would you describe your child’s weight at the moment?’ (underweight, healthy weight, overweight, very overweight) [39].
- visual analogue scale which consists of a 10 cm line with ends labelled ‘extremely underweight’ and ‘extremely overweight’. Respondents mark the spot on the line indicating how they perceive their child’s weight [40].

#### Parent behaviours

Parent knowledge of the health consequences of childhood overweight, intention to change their child lifestyle behaviours (diet and physical activity) and/or seek advice/support, self-efficacy/perceived control over child’s behaviour, action planning, coping planning [23, 26, 27, 36] will be assessed. After a definition of a ‘healthy diet’ there are questions on intention (1 item; “I intend to support my child to have a healthy diet during the next month”), perceived control over child’s behaviour (1 item: how much control do you have over your child’s diet during the next month?), self-efficacy (1 item; “I believe that I can support my child to have a healthy diet during the next month”), action planning (3 items; for example “During the next month I have a detailed plan on what healthy diet my child will eat”) and coping planning (5 items; for example “During the next month I have a detailed plan on what to do if something interferes with my plans to support my child’s healthy diet”). The same constructs are assessed targeting two other relevant behaviours: supporting their children in being physically active and seeking support for their child’s weight. For these two behaviours similar procedures are used: first presenting a definition of the target behaviour, and then asking parents to answer the theory driven items.

#### Anthropometry

- Child’s height and weight at baseline
- Child’s height and weight at 12 month follow-up
- Parents’ weight status based on self-reported height and weight measures

#### Demographic measures

- Child’s sex
- Family residential postcode

### Sample size

The required sample size is based on detecting a difference in a dichotomous outcome. There are two intervention groups and a control group; the primary comparisons of interest will be between each of the intervention groups and the control group. To take into account the multiple comparisons, the type 1 error rate is specified as 2.5 %. The unit of randomisation is the school.

We estimate the number of eligible children per school completing the study to be 15. This is based on the assumptions that we will survey 120 children per school (2 classes of 30 in each of the two year groups). Of these children we anticipate that 40 (33 %) will meet the study analysis entry criteria (i.e. be overweight) and that of these 20 (50 %) will agree to participate and that of these we will be able to collect follow up data at 12 months for 15 (75 %).

The study is estimated to have 90 % power to detect a 20 point difference between the control group and each of the intervention groups at one month (that is, 30 % of parents accurately perceiving their overweight child as overweight versus 50 %). Applying Fleiss’s method for a proportion incorporating a continuity correction, we would need a final sample size of 471 overweight children (three groups of 157). With an assumed intra-cluster correlation coefficient of 0.01 and a mean cluster size of 15, the design effect is 1 + 14*0.01 = 1.14 implying that we need a final sample size of at least 537 children and thus we will need a minimum of 36 schools. With 120 children per school this will provide a sampling frame of 4320 children. The target sample size is 2160 parent–child pairs.

### Statistical analysis

Analysis will be restricted to the parents of the overweight children. For the primary outcome measure (dichotomous variable based on correct/incorrect perception of weight status) the null hypothesis is that there is no difference in the proportion of parents with the correct perception of child body weight status between individuals in the control or intervention groups. This will be tested using a logistic multilevel model to account for clustering at school level using specialist statistical software such as MLwiN [41]. Using a multilevel modelling approach will also allow variation in change in perception to be partitioned in to school, year group and individual levels and will permit the inclusion of characteristics of individuals (IMD) and schools (Free School Meal Index and IMD) and anthropometric measures for both child and parent. Inclusion of such terms may reduce the estimate of the standard deviation for the effect and hence provide a more precise estimate of the effect of the intervention. A continuous variable of perception of the child’s weight based on the VAS is also a primary outcome measure.

The secondary outcome measures include knowledge of the consequences of childhood overweight, intention to change lifestyle behaviours and/or seek advice, perceived control, action planning, coping planning, seeking support behaviours, and the child’s body weight status at 12 month follow-up. Process evaluation will use the same statistical approach to test group differences on any of the psychological secondary outcomes.

Although a formal economic analysis is not part of this trial, the study team will work with health economists and use our existing knowledge of the literature to consider the economic implications of our findings.

## Discussion

Given the increasing prevalence of childhood overweight, it is important to take preventive public health action. Parents play a crucial role in shaping health-related behaviours but they will not take action for overweight in their child if they do not recognise it. It has previously been shown that parents do not well understand how to plot their child’s BMI onto growth charts, but that they do use visual cues to identify their child’s weight status [6, 14]. This suggests the need to develop and test intervention strategies which tap into this tendency to rely on visual methods. This paper presents the protocol for a trial to test whether the newly developed Map Me intervention is effective in improving parents’ perception of overweight in their child. If it is effective, it has potential to be included in public health interventions aimed at parents, with possible health benefits to children at a population level. It will provide a unique opportunity to help parents recognise when their child is overweight and why taking appropriate action is important. Given the evidence about parents’ lack of acceptance of the NCMP results [9, 10], Map Me might provide an effective resource to support health care professionals’ feedback to parents and facilitate communication with parents about child overweight. Furthermore, NHS Choices (the online service provided by the NHS), which is aimed at the general population, is in an ideal position to use Map Me on its website. NHS Choices is a much used site; the online ‘BMI healthy weight calculator’ for one year (July 2012 to June 2013) was visited 8,239,750 times, and the App for measuring BMI was downloaded 306,204 times. Although website development costs may be relatively high and there are associated on-going maintenance costs with them, these costs do not change according to the numbers visiting the website. The benefit of the website is therefore potentially cost-effective. Map Me might also be a useful resource for health care professionals as they too are generally poor at assessing the weight status of children and tend to underestimate overweight and obesity in children [42].

In conclusion, Map Me is a potentially valuable research and educational resource. It presents a potentially important contribution to the field of childhood overweight prevention, providing benefits to children, families, health professionals, researchers, and wider society.

## Abbreviations

NCMP: National Child Measurement Programme
BMI: Body mass index
NHS: National Health Service
LEA: Local Education Authorities
IMD: ndex of Multiple Deprivation
